# Ergotine in Hemorrhage

**Published:** 1846-12

**Authors:** 

**Affiliations:** Chambery


*Ergotine in Hemorrhage, by M. Bonjean, of Chambery.*—From the ergot of rye M. Bonjean has extracted two substances, differing very materially from each other in their nature and in their properties. The first is a fixed oil, in which seem to reside the poisonous elements of the ergot; the other is ergotine, a watery extract, to which M. Bonjean attributes considerable hemostatic virtues. This opinion is also common to M. Müller and Dr. Wright; but hitherto its correctness has been tested only on animals, and in them the great plasticity of the blood diminishes the value of the experiments. M. Bonjean has forwarded to the Academy a case of hemorrhage arrested by the ergotine in the human subject:—A stout woman, aged forty, wounded her hand, on the 5th of June, with the fragments of a glass bottle: a branch of the palmar artery was divided and profuse hemorrhage ensued, against which compression was of no avail. In the space of two minutes the application of the concentrated ergotine stopped the flow of blood, which did not afterwards recur.—*London Medical Times.*

